# Dimethyl Fumarate Strongly Ameliorates Gray and White Matter Brain Injury and Modulates Glial Activation after Severe Hypoxia–Ischemia in Neonatal Rats

**DOI:** 10.3390/antiox13091122

**Published:** 2024-09-16

**Authors:** Jon Ander Alart, Antonia Álvarez, Ana Catalan, Borja Herrero de la Parte, Daniel Alonso-Alconada

**Affiliations:** 1 Department of Cell Biology and Histology, School of Medicine and Nursing, University of the Basque Country (UPV/EHU), 48940 Leioa, Spain; jonander.alart@ehu.eus (J.A.A.); antoniaangeles.alvarez@ehu.eus (A.Á.); 2 Psychiatry Department, OSI Bilbao-Basurto, Basurto University Hospital, 48013 Bilbao, Spain; ana.catalanalcantara@osakidetza.eus; 3 Neuroscience Department, University of the Basque Country (UPV/EHU), 48013 Leioa, Spain; 4 Biobizkaia Health Research Institute, 48903 Barakaldo, Spain; 5 CIBERSAM, Centro Investigación Biomédica en Red de Salud Mental, 28007 Madrid, Spain; 6 Department of Psychosis Studies, Institute of Psychiatry, Psychology and Neuroscience, King’s College London, London SE5 8AF, UK; 7 Department of Surgery and Radiology and Physical Medicine, School of Medicine and Nursing, University of the Basque Country (UPV/EHU), 48940 Leioa, Spain; borja.herrero@ehu.eus

**Keywords:** neonatal brain injury, hypoxia–ischemia, dimethyl fumarate, neuroprotection, white matter injury, microglia, astroglia

## Abstract

Neonatal hypoxia–ischemia is a major cause of infant death and disability. The only clinically accepted treatment is therapeutic hypothermia; however, cooling is less effective in the most severely encephalopathic infants. Here, we wanted to test the neuroprotective effect of the antioxidant dimethyl fumarate after severe hypoxia–ischemia in neonatal rats. We used a modified Rice–Vannucci model to generate severe hypoxic–ischemic brain damage in day 7 postnatal rats, which were randomized into four experimental groups: Sham, Sham + DMF, non-treated HI, and HI + DMF. We analyzed brain tissue loss, global and regional (cortex and hippocampus) neuropathological scores, white matter injury, and microglial and astroglial reactivity. Compared to non-treated HI animals, HI + DMF pups showed a reduced brain area loss (*p* = 0.0031), an improved neuropathological score (*p* = 0.0016), reduced white matter injuries by preserving myelin tracts (*p* < 0.001), and diminished astroglial (*p* < 0.001) and microglial (*p* < 0.01) activation. After severe hypoxia–ischemia in neonatal rats, DMF induced a strong neuroprotective response, reducing cerebral infarction, gray and white matter damage, and astroglial and microglial activation. Although further molecular studies are needed and its translation to human babies would need to evaluate the molecule in piglets or lambs, DMF may be a potential treatment against neonatal encephalopathy.

## 1. Introduction

Neonatal hypoxia–ischemia (HI), caused by lack of oxygen and/or blood flow to the brain of the near-term neonate [1], is a major cause of death and disability in infants [2,3]. Its incidence is relatively high, with 3 to 5 out of 1000 newborns suffering from HI and around 20% of these infants developing severe neurological consequences such as hypoxic–ischemic encephalopathy [4]. Subsequent conditions may include disorders such as sensory or cognitive impairment, epilepsy, or cerebral palsy [5].

The only standardized treatment at present is moderate hypothermia, which consists of reducing the brain temperature to 33.5 °C in a controlled way for 72 h [6]. The benefits of cooling are limited, as it only reduces the risk of death and sequelae by 12%, leaving a prevalence of 50% [7]. Thus, a large number of affected neonates will suffer from neurological disorders despite the implementation of this treatment [2,8,9]. Further, meta-analysis of randomized clinical trials has shown that its therapeutic effect is less favorable in severe cases [2]; a lesser benefit was also observed in preclinical studies [10] after severe HI.

The brain is the most metabolically active organ, and it also has a fragile redox homeostasis, with low antioxidant defenses and a high content of easily oxidizable membrane lipids. In standard physiological situations, the antioxidant system effectively prevents cell damage; however, in situations such as perinatal asphyxia, it may fail. After HI, one of the key factors determining neural damage and cell death is the pathological overproduction of reactive oxygen species and subsequent oxidative stress [11]. Moreover, the newborn brain is especially susceptible to oxidative stress due to an abundance of unsaturated fatty acids, high oxygen consumption, and an underdeveloped antioxidant system with limited production of antioxidant enzymes, among others [12]. Oxidative stress is also linked to the activation of glial cells after neonatal brain injury. Activated astrocytes and microglia produce pro-inflammatory molecules and additional reactive oxygen species, which further increase neuronal death (gray matter injury) [13] and disrupt cerebral myelination (white matter damage) [14].

Given the critical role of cellular redox status in the newborn brain and its functions as a modulator of the pathological responses in cases of HI, antioxidant agents are proposed as an effective treatment for this pathology [15]. Dimethyl fumarate (DMF) is an ester of fumaric acid [16] with strong antioxidant [17] and anti-inflammatory properties [18]. Its immunomodulatory capacity has been tested against polymicrobial sepsis [19], while its neuroprotective effects have been demonstrated in Alzheimer’s disease [20]. Furthermore, DMF has shown efficacy in reducing microgliosis and white matter injury following chronic cerebral hypoperfusion [21], traumatic brain injury [22], and ischemia [23] in adult rodents.

To our knowledge, its potential neuroprotective effect has not been tested yet in neonatal models of brain injury. Given the limitations of therapeutic hypothermia and the need to find alternative therapies to treat severe cases of neonatal brain injury, we aimed to test the neuroprotective capacity of DMF in a relevant preclinical model of neonatal HI. Specifically, we assessed cerebral infarction, global and regional neuropathological status of the brain, white matter injury, and microgliosis and astrogliosis in neonatal rats subjected to severe HI and treated with DMF.

## 2. Materials and Methods

All experimentation was regulated and approved by the Committee on the Ethics of Animal Experiments of the University of the Basque Country (UPV/EHU) (Ref. M20/2023/153), based on the regulations in application and development of Law 6/2013 and RD53/2013.

### 2.1. Hypoxia–Ischemia (HI)

Experiments were conducted on Sprague–Dawley neonatal rats. Pregnant rats were housed in individual cages and provided with food and water ad libitum, under a 12 h:12 h light/dark cycle. On the day of delivery, the litters were standardized to 10 individuals. Seven days later, on postnatal day 7 (PD7), rats were randomly distributed into four experimental groups (see below). Severe HI was induced following a modified protocol of the commonly used Rice–Vannucci model of neonatal HI. Briefly, neonatal rats were anesthetized via isoflurane inhalation (4% induction, 1.5% maintenance), and the left common carotid artery was double ligated and cauterized. After completion of the ischemic procedure (which lasted no more than 10 min), the pups were returned with their dams for one hour to recover from surgery and anesthesia. To induce hypoxia, pups were placed in hermetically sealed chambers and exposed to a gaseous mixture of 92%N_2_/8%O_2_, with a gas flow rate of 6 L/min. The hypoxic chambers were kept in a hot water bath to maintain pups’ body temperature at 37 °C. Hypoxic exposure was extended to 150 min to cause severe brain damage [10]. 

At the end of the hypoxia, animals were randomly assigned to the following experimental groups:

- Sham (n = 13): pups without ischemia or hypoxia.
- Sham + DMF (n = 10): pups without ischemia or hypoxia receiving DMF.
- HI (n = 24): hypoxic–ischemic non-treated pups.
- HI + DMF (n = 13): hypoxic–ischemic pups receiving DMF.

### 2.2. DMF Administration

DMF (Sigma-Aldrich, Burlington, MA, USA; #242926) was administered at a concentration of 45 mg/kg diluted in 10% of dimethyl sulfoxide (DMSO) (Sigma-Aldrich, #D2650) and 90% of 0.1 M phosphate-buffered saline (PBS) (Gibco, Waltham, MA, USA; #70011-036).

The first dose was administered immediately after the hypoxia process via intraperitoneal injection; 100 µL of the above solution was administered to each animal. After 12 h, the first oral dose of the treatment (same concentration and volume) was administered using cannulas designed for this purpose. These oral doses were administered 5 more times, every 12 h, for a total of 6 oral doses per animal [23].

### 2.3. Obtention and Processing of Samples

Seven days after HI (postnatal day 14, PD14), rat pups were sacrificed by intraperitoneal injection of sodium pentobarbital (200 mg; 100 μL) and perfusion fixed with 4% paraformaldehyde diluted in PBS (0.1 mol/L, pH 7.2–7.4). Brains were removed and kept in the same fixative at 4 °C overnight. The next day, brains were sectioned into 4 mm coronal slices using a standard rat brain matrix and embedded in paraffin. Using a microtome, 5 µm slices were obtained at the level of mid-dorsal hippocampus and thalamus (Bregma—1.80 mm) according to Khazipov et al. [24].

### 2.4. Brain Injury Assessment 

Using hematoxylin–eosin-stained samples, a neuropathological score was obtained with the help of an Olympus BX50 light microscope according to Beldarrain [25]. Histological damage was analyzed both globally (total) and regionally (parietal cortex and hippocampus) as follows:

First, we assessed macroscopic damage: 0 = no observable injury; 3 = observable cortical injury. Then, microscopic damage was evaluated in the parietal cortex: 0 = no observable injury; 2 = a few small, isolated groups of injured cells; 4 = several larger groups of injured cells, mild infarction; 7 = moderate confluent infarction; 9 = extensive confluent infarction. By adding macroscopic damage, parietal cortex scores can range from 0 (no damage) to 12 (maximum punctuation of damage).

Finally, hippocampal damage was evaluated in CA-1, CA2/3, and dentate gyrus sub-regions as follows: 0 = no observable injury; 1 = mild infarction; 2 = moderate infarction; 3 = severe infarction. Total hippocampal damage was obtained by adding the scores from these 3 sub-regions, thus ranging from 0 (no damage) to 9 (maximum punctuation of damage).

Global/total score was graded from 0 (no damage) to 21 (maximum punctuation of damage) by adding the values of parietal cortex affectation (ranging from 0 to 12) and hippocampal damage (ranging from 0 to 9).

The evaluation of these parameters was performed by two histologists blind to the experimental conditions, and data were analyzed by using a Kruskal–Wallis test with Dunn’s multiple comparison test.

Further, the ratios of the ipsilateral-to-contralateral hemispheric and hippocampal areas were obtained to determine tissue loss using an image analysis program (Fiji-ImageJ 1.53). To carry out this measurement, the areas in pixels of the ipsilateral and contralateral hemispheres and hippocampi were measured. In hematoxylin–eosin-stained samples, cortical and hippocampal infarcted area is usually lost (in severe cases) or shows a reduction/loss of the staining pattern (mild-to-moderate damage), visible even at lower magnification.

We thus measured the tissue area that did not suffer from this loss of staining. After measuring ipsilateral (damaged) hemispheres and hippocampi, we divided these values by contralateral (non-damaged) hemispheric and hippocampal tissue areas, thus obtaining the ratios from each animal and experimental condition. In the case of no damage, the ratio values would be close to 1, since infarcted animals will show a reduction in ratios. These data were analyzed using a Kruskal–Wallis test with a Dunn’s multiple comparison test.

### 2.5. Immunohistochemistry

Brain samples for immunohistochemistry were deparaffinized and hydrated, and then immersed in a sodium citrate solution for antigen retrieval (10 mM sodium citrate + 0.05% Tween20 in distilled water at pH 6) and boiled 3 times. Once cooled to room temperature, slices were washed with distilled water and endogenous peroxidase blocked with 3% H_2_O_2_ and PBS for half an hour. After washing, samples were incubated for 1 h in blocking buffer (5% bovine serum albumin + 0.4% Triton X-100 in PBS). Following another wash, samples were incubated at 4 °C overnight with primary antibodies to myelin basic protein (MBP, 1:500; Abcam, Cambridge, UK; #ab65988), glial fibrillary acidic protein (GFAP, 1:100; Invitrogen, Waltham, MA, USA; #MA5-12023), and ionized calcium-binding adapter molecule 1 (Iba-1, 1:500; Palex Medical, Barcelona, Spain; #517917). The next day, slices were washed with PBS and incubated for one hour with their secondary antibodies at room temperature (goat anti-rabbit IgG, #65-6140; goat anti-mouse IgG, #31800; Invitrogen, Thermo Fischer, Waltham, MA, USA), followed by three washes in PBS, an incubation with horseradish peroxidase-streptavidin conjugate (1:500, #43-4323, Thermo Fisher) for 30 min and later diaminobenzidine-revealed (DAB). Finally, samples were counterstained using hematoxylin and mounted with dibutylphthalate polystyrene xylene.

### 2.6. White Matter Injury Assessment

**Figure 1:**
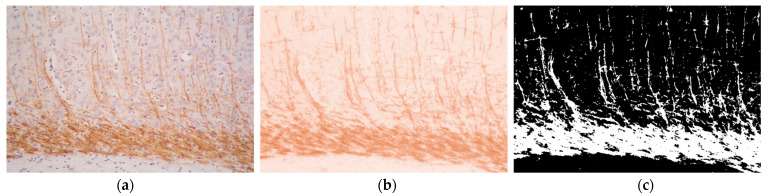
Image analysis process used to quantify MBP expression as a subrogate of white matter injury: (**a**) original microphtograph stained with MBP immunohistochemistry; (**b**) color deconvolution photograph to retain brown color; (**c**) black and white binarized image after threshold.

White matter injury was assessed with MBP quantification using the image analysis software Fiji-ImageJ v.1.53 (Figure 1). Photographs were taken at the level of mid-dorsal hippocampus and thalamus (Bregma—1.80 mm) in three brain regions: the cingulum, the external capsule, and the caudoputamen at 200× magnification. Color deconvolution was applied to the images to isolate the brown color, as the main DAB signal color. Using the original photograph as a reference in order to avoid loss or excess of DAB signal, a threshold was then performed to binarize the image to black (negative or non-stained) and white (positive or stained) pixels, the latter used for quantification. This process was performed on both the left/ipsilateral (damaged) and right/contralateral (non-damaged) hemispheres, then the left-to-right (L:R) ratios were calculated. Brain samples with absent white matter lesion have ratios close to 1, whereas MBP loss is reflected in reduced ratios. These data were analyzed using a Kruskal–Wallis test with Dunn’s multiple comparison test.

### 2.7. GFAP and Iba-1 Immunoreactivity

Brain samples were scanned with a high-resolution camera (Slide Scanner 3D Histech Pannoramic Midi II), obtaining a panoramic scan of the brain at the mid-dorsal hippocampus and thalamus. Using an image analysis program (SlideViewer 2.7, 3DHISTECH), the areas of interest were selected (hippocampus, somatosensory cortex, perirhinal cortex, and striatum) from both hemispheres, creating new images from the original scan. These new images were converted from MRXS format to TIFF format using the Slide Converter 2.3.2, 3DHISTECH software. The images were then analyzed again with Fiji-ImageJ 1.53. Similar to the MBP evaluation, the quantification of GFAP (astrocytes) and Iba-1 (microglia) immunostaining pattern was performed using color deconvolution followed by threshold, and data were obtained as positive pixels/µm^2^.

### 2.8. Statistical Analyses

A two-tailed, unpaired Student’s t-test or one-way ANOVA (with Tukey’s multiple comparison test) was performed for comparisons of parametric data; non-parametric data were analyzed with Mann–Whitney or Kruskal–Wallis tests (with Dunn’s multiple comparison test). Bar graphs appear as mean ± standard deviation (SD). Statistical analysis was performed using the GraphPad Prism 10 software package (GraphPad Software, Inc., La Jolla, CA, USA). Data were considered significantly different if *p* < 0.05.

## 3. Results

No pups died in the Sham or Sham + DMF groups. In the HI group, two pups (n = 2) died during hypoxia. In the HI + DMF group, one pup died during hypoxia (n = 1) and another pup died on postnatal day 11 (n = 1).

### 3.1. Neuropathological Score

**Figure 2:**
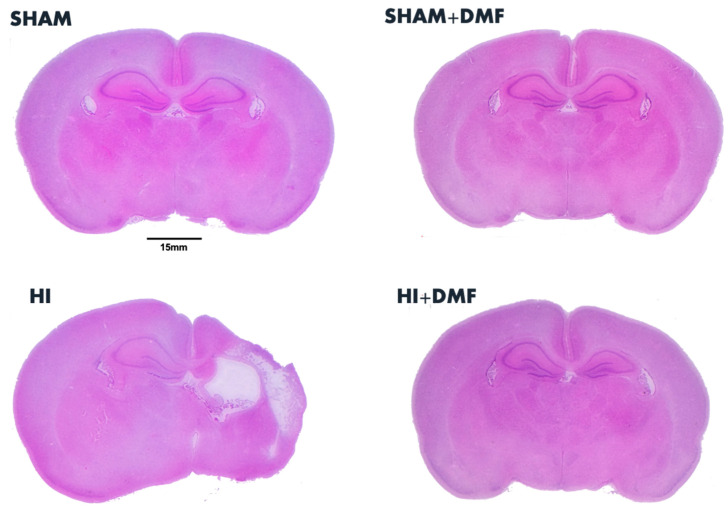
Representative images of brain sections obtained at the level of mid-dorsal hippocampus and thalamus stained with hematoxylin–eosin from Sham, Sham + DMF, non-treated HI, and HI + DMF groups.

**Figure 3:**
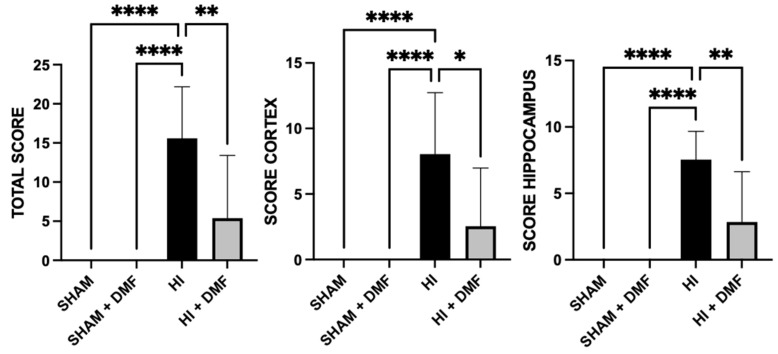
Global (total) and regional (cortex and hippocampus) neuropathological score values from Sham, Sham + DMF, non-treated HI, and HI + DMF groups. Maximum score values (and damage) for global, cortex, and hippocampus are 21, 12, and 9, respectively. Data were analyzed using a Kruskal–Wallis test with Dunn’s multiple comparison test. * *p* < 0.05; ** *p* < 0.01; **** *p* < 0.0001 vs. HI.

Figure 2 (microphotographs) and 3 (graphs) show the differences in the neuropathological score from Sham, Sham + DMF, HI, and HI + DMF animals. There were no signs of neurotoxicity, brain damage, or edema in Sham + DMF animals, whose neuropathological scores were 0, the same as for the Sham group (Figure 3).

We first performed a histopathological analysis (detailed in Section 2.4) of the whole slice (total score), which revealed significant differences between non-treated and DMF-treated HI animals (Figure 3). Non-treated HI animals showed a high neuropathological score (15.583), significantly higher than Sham groups (*p* = 0.0001). Pups treated with the antioxidant DMF (HI + DMF) revealed a significant reduction in global neuropathological score (5.385) when compared to HI (*p* = 0.0016), being similar to the Sham group.

Additionally, brain damage was evaluated regionally (Figure 3), with the cortex of non-treated HI pups showing an elevated score (8.042), significantly higher than both Sham (0, *p* = 0.0001) and HI + DMF (2.538, *p* = 0.0138) groups, revealing DMF’s neuroprotection in this region. We also evaluated the hippocampus as a brain region of special vulnerability to HI lesions, obtaining similar results: the non-treated HI group had greater histological damage (7.542) compared to the Sham group (0, *p* = 0.0001) as well as to animals treated with DMF (2.846, *p* = 0.0068). Consequently, all areas studied showed reduced histological damage (reduced cell death and small or absent infarct zones) in animals treated with the antioxidant DMF, obtaining similar values to the Sham group.

### 3.2. Brain Area Loss

**Figure 4:**
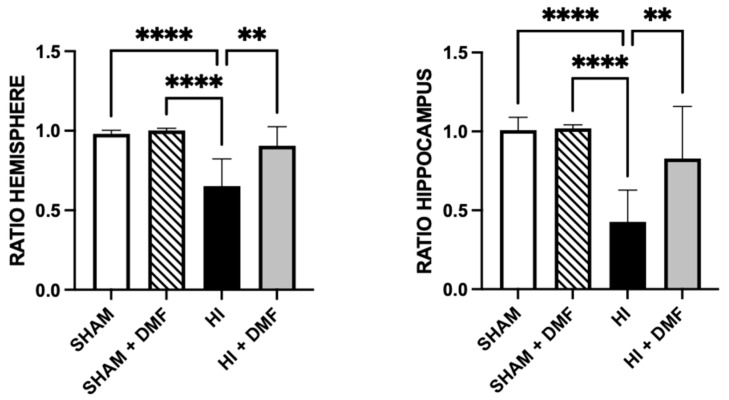
Hemispheric and hippocampal left-to-right area ratios from Sham, Sham + DMF, non-treated HI, and HI + DMF groups. Maximum ratio value is 1. Data were analyzed using a Kruskal–Wallis test with Dunn’s multiple comparison test. ** *p* < 0.01; **** *p* < 0.0001 vs. HI.

Figure 4 shows the cerebral infarct/tissue loss as the ratio of the ipsilateral to the contralateral hemisphere, as detailed in Section 2.4. There were no signs of neurotoxicity in Sham animals receiving DMF (ratio: 1.002), as hemispheric ratio was similar to Sham (0.990).

Non-treated HI pups (0.652) showed the greatest tissue loss when compared to Sham pups (*p* = 0.0001). Animals treated with DMF, however, showed higher hemispheric ratios (0.906) when compared to non-treated pups (*p* = 0.0031), obtaining similar values to Sham. The same analysis was performed in the hippocampus, obtaining similar results: non-treated HI animals (0.427) showed a greater loss of hippocampal tissue than Sham animals (1.008; *p* = 0.0001). HI animals treated with DMF (0.828) showed a strong reduction in area loss (*p* = 0.007), obtaining similar values to Sham (Figure 4).

### 3.3. Myelin Basic Protein Quantification

**Figure 5:**
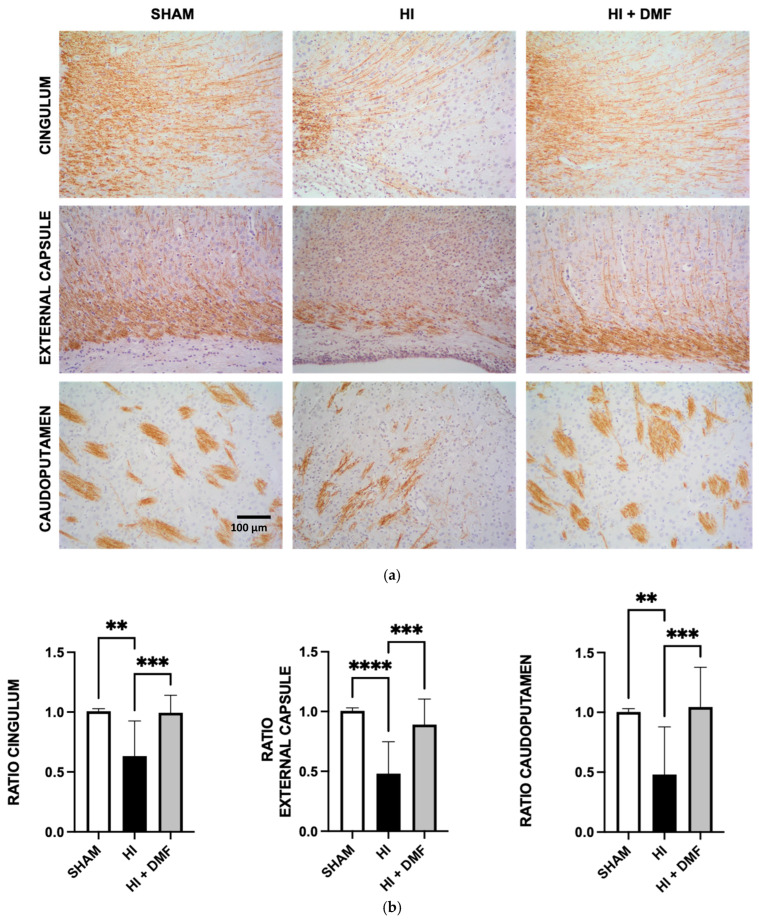
(**a**) Representative images of brain sections at the level of cingulum, external capsule, and caudoputamen stained with MBP immunohistochemistry stained in brown from Sham, non-treated HI, and HI + DMF groups; Scale bar corresponds to 100 µm. (**b**) comparison of the ipsilateral hemisphere with the contralateral hemisphere (L:R) in ratios in regional (cingulum, external capsule, and caudoputamen) sections from Sham, non-treated HI, and HI + DMF groups. Maximum ratio value is 1. Data were analyzed using a Kruskal–Wallis test with Dunn’s multiple comparison test. ** *p* < 0.01; *** *p* < 0.001; **** *p* < 0.0001 vs. HI.

White matter tracts were analyzed by evaluating MBP expression patterns in three brain regions (cingulum, external capsule, and caudoputamen) from both hemispheres (detailed in Section 2.6). Data were obtained as the left = ipsilateral-to-right = contralateral (L:R) ratio of MBP expression; the results are shown at the bottom of Figure 5.

We found significant differences in the three brain areas analyzed between non-treated HI rats and the other experimental groups. In the cingulum, HI pups showed a reduced MBP ratio (0.632) compared to Sham (1.008; *p* = 0.0063) and DMF-treated (0.995; *p* = 0.0007) rats. The same response was observed in the external capsule, with a higher MBP ratio in Sham (1.006; *p* = 0.0001) and DMF (0.889; *p* = 0.0005) animals when compared to non-treated HI pups (0.481). The caudoputamen also revealed non-treated HI animals with reduced L/R MBP ratio (0.480), with significant differences when compared to Sham (1.003; *p* = 0.0031) and DMF-treated (1.045; *p* = 0.0002) rats. No significant differences were found between the Sham group and the HI + DMF group, both having similar MBP values (Figure 5b).

### 3.4. GFAP Immunoreactivity

**Figure 6:**
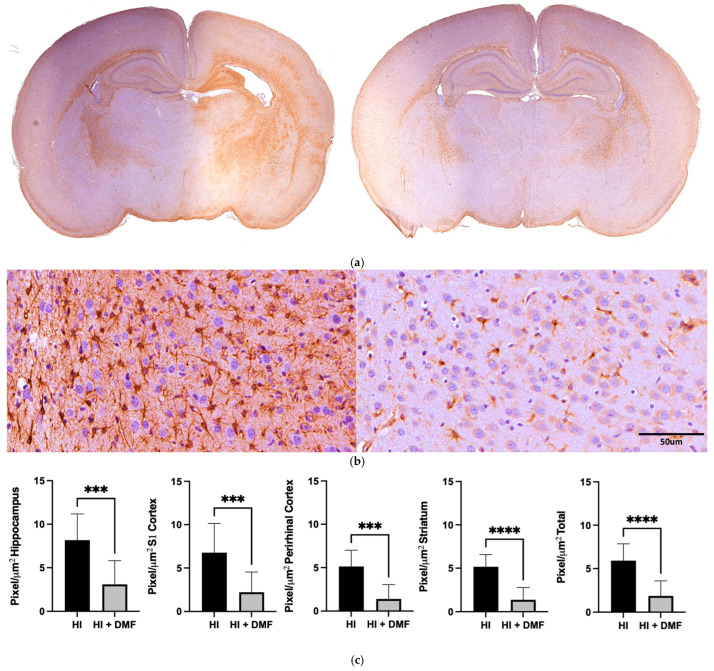
(**a**) Low-magnification representative photographs of brain sections obtained at the level of mid-dorsal hippocampus and thalamus stained with GFAP immunohistochemistry from non-treated HI and HI + DMF groups; (**b**) high-magnification representative microphotographs of the morphology of GFAP-labeled astrocytic cells, showing their cytoplasm (and cellular projections) stained in brown, whereas non-astrocytic cells appear purple/blue due to hematoxylin counterstain; (**c**) regional (hippocampus, S1 cortex, perirhinal cortex, and striatum) and global (total) comparison of the amount of GFAP in pixels/µm^2^ from non-treated HI and HI + DMF groups. Data were analyzed using a Mann–Whitney test. *** *p* < 0.001; **** *p* < 0.0001 vs. HI.

Figure 6 shows the immunohistochemical differences in astroglial staining pattern between the HI group and the HI + DMF-treated group after GFAP labeling. Figure 6b presents two photographs showing GFAP-positive cells corresponding to astrocytes; their cytoplasm and projections appear brown, while non-astrocytic cells appear stained blue/purple as a result of the hematoxylin counterstain. The response of astroglia between the different groups was studied in four different brain areas (hippocampus, somatosensory/S1 cortex, perirhinal cortex, and striatum) and in the whole hemisphere (total). 

In all areas analyzed, there was a significant decrease in GFAP immunolabeling after the treatment with DMF: hippocampus (HI: 8.175 vs. HI + DMF: 3.089; *p* = 0.0006), S1 cortex (HI: 6.771 vs. HI + DMF: 2.223; *p* = 0.0003), perirhinal cortex (HI: 5.137 vs. HI + DMF: 1.396; *p* = 0.0001), striatum (HI: 5.169 vs. HI + DMF: 1.368; *p* = 0.0001), and whole hemisphere (HI: 5.921 vs. HI + DMF: 1.872; *p* = 0.0001) (Figure 6c). The decrease in GFAP labeling in DMF-treated animals suggests that, after hypoxic–ischemic events, the treatment is able to control the astroglial response to injury, which usually corresponds to higher numbers of astrocytes and/or longer cytoplasmic projections, as can be observed in comparison to the contralateral hemispheres (Figure 6a).

### 3.5. Iba-1 Immunoreactivity

**Figure 7:**
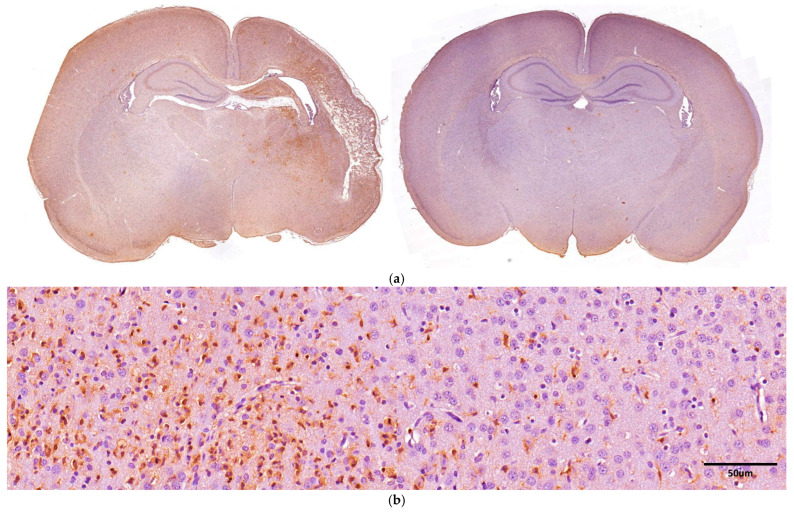

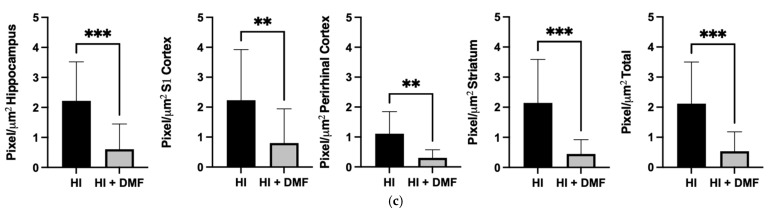
(**a**) Low-magnification representative images of brain sections obtained at the level of mid-dorsal hippocampus and thalamus immunostained with Iba-1 from non-treated HI (left photograph) and HI + DMF (right photograph) groups; (**b**) high-magnification representative microphotographs of the morphology of Iba-1-labeled microglial cells, showing their cytoplasm stained in brown, whereas non-microglial cells appear purple/blue due to hematoxylin counterstain; (**c**) regional (hippocampus, S1 cortex, perirhinal cortex, and striatum) and global (total) comparison of the amount of Iba-1 in pixels/µm^2^ from non-treated HI and HI + DMF groups. Data were analyzed using a Mann–Whitney test. ** *p* < 0.01; *** *p* < 0.001 vs. HI.

Figure 7 shows the immunohistochemical differences in the microglial marker Iba-1 between the HI and the HI + DMF-treated groups. Iba-1 positive cells (corresponding to macrophages/microglia) have been labeled using DAB, thus staining their cytoplasm with a brown color, while the other cells appear blue/purple due to the hematoxylin counterstain. As for astrocytes, the microglial response was also evaluated regionally (in hippocampus, somato-sensory/S1 cortex, perirhinal cortex, and striatum) and in the whole hemisphere (total). 

Similar to GFAP, Iba-1 immunohistochemistry revealed a significant decrease in Iba-1 expression after the treatment with DMF in all areas analyzed: hippocampus (HI: 2.217 vs. HI + DMF: 0.606; *p* = 0.0007), S1 cortex (HI: 2.232 vs. HI + DMF: 0.804; *p* = 0.0013), perirhinal cortex (HI: 1.110 vs. HI + DMF: 0.302; *p* = 0.0019), striatum (HI: 2.145 vs. HI + DMF: 0.446; *p* = 0.0002), and the whole hemisphere (HI: 2.117 vs. HI + DMF: 0.537; *p* = 0.0004) (Figure 7c). The difference in Iba-1 labeling between non-treated HI and HI + DMF-treated animals suggests that DMF may reduce microgliosis after hypoxic–ischemic injury, as non-treated animals showed relevant signs of activated of microglia when compared to their contralateral hemisphere (Figure 7a).

## 4. Discussion

In this study, DMF showed strong neuroprotective effects in a preclinical model of severe HI in newborn rats. DMF was able to protect the neonatal rat brain by preserving brain tissue (reducing both gray and white matter damage) and by modulating astroglial and microglial activation.

Therapeutic hypothermia has become the standard treatment for neonatal HI; however, clinical trials [26] and meta-analyses [2] have suggested that cooling may not be protective in the most severely encephalopathic infants. This was also observed in preclinical models of neonatal HI [10], where immediate hypothermia was not protective, and delayed cooling was even detrimental after severe brain injury. Therefore, alternative treatments for infants with severe encephalopathy are urgently needed.

Here, we used the Rice–Vannucci preclinical model of neonatal HI in rats, extending the hypoxia duration to 150 min to induce severe brain damage [10]. This protocol resulted in severe histological damage in non-treated HI animals, with significant brain tissue loss in the ipsilateral (damaged) hemisphere, as previously reported [10,27]. This was further confirmed by the neuropathological score, revealing substantial global and regional (hippocampal and cortical) neuronal damage to gray matter. Conversely, DMF-treated animals exhibited minimal brain tissue loss and significantly reduced gray matter injury, even approaching values observed in sham-operated controls. These findings suggest a potent neuroprotective effect of DMF following severe neonatal brain injury.

In a recent study [28], DMF was not able to reduce neurological deficit scores in adult rats subjected to middle cerebral artery occlusion, but the animals were euthanized only at 72 h after injury, in the sub-acute phase, and DMF also revealed to reduce neuronal swelling and cell death. Consistent with our data, other studies using adult mice [23] and rats [29] have described strong neuroprotection after DMF treatment, reducing cerebral infarct volume and improving the histological status of the brain.

As a common feature after perinatal asphyxia and subsequent hypoxic–ischemic encephalopathy, we also analyzed white matter injury. Research has identified various histological characteristics in the white matter, such as cell death, edema, gliosis, and decreased myelination [14,30], which are associated with cognitive impairment in children [31,32]. Myelin deficiency can lead to negative impacts on axonal function and neuronal survival, which manifests as abnormal nerve impulse transmission, ultimately causing long-lasting neurodevelopmental issues [33].

A decrease in MBP staining (the primary myelin protein) is recognized as a characteristic sign of reduced myelin production and white matter injury [34,35]. In this study, densitometry analysis revealed a significant loss in MBP immunostaining caused by HI in the three brain regions evaluated (cingulum, external capsule, and caudoputamen). However, treatment with DMF maintained myelination levels similar to the Sham group in all brain areas analyzed. This protective effect of DMF on white matter has been also observed in mouse models of multiple sclerosis, where the compound showed potential to suppress demyelination and axonal loss by quantifying MBP [36].

In the neonatal brain, white matter injury may occur when immature oligodendrocytes are damaged, impeding their differentiation into mature oligodendrocytes that carry out myelination [14]. The mechanism of damage of oligodendrocytes usually converges on three interacting processes: excitotoxicity, overproduction of free radicals, and astroglial and microglial activation [37,38]. Here, alongside reduced myelination, the densitometric analysis of GFAP (as a subrogate of astroglial activation) and Iba-1 (a specific marker of microglia) revealed that severe neonatal HI induced the activation of both astrocytes and microglial cells, which in turn may develop white matter damage. These results align with those obtained in previous studies by us [39,40] and others [14,41] evaluating reactive gliosis after neonatal brain injury.

DMF is considered an effective modulator of glial cells and may be promising in combatting brain inflammation and neurodegeneration [42]. In this study, DMF administration led to a reduction in both GFAP and Iba-1 densitometric values, suggesting that the compound has the capacity to regulate the activation of both astrocytes and microglia following severe HI. In vitro, DMF significantly decreased the production of proinflammatory mediators in classically activated microglia and additionally alleviated mitochondrial respiratory deficiencies in primary cortical neurons [43]. In vivo, DMF also demonstrated potent immunomodulatory effects after stroke in adult rats, reducing neutrophil and T cell infiltration, as well as the number of activated microglia/macrophages in the infarct region [44]. 

Here, we did not study the effect of DMF at the molecular level, but other studies using adult rodent stroke models can serve as a basis for understanding some of its mechanisms of action. In mice, DMF activates the redox-sensitive transcription factor NF-E2-related factor 2 (Nrf2) signaling pathway in astrocytes [45], which in turn strengthens the antioxidant and anti-inflammatory system [46] and stabilizes the blood–brain barrier [45]. The relationship between DMF and Nrf2 was further confirmed using wild-type and knockout mice for this transcription factor. After DMF treatment, wild-type mice showed increased Nrf2 expression, followed by reduced infarct volume, edema, and neuronal death, and ameliorated reactive gliosis. This effect on glial cells was similar to that described here for neonatal rats, as DMF administration resulted in decreased activation of microglia and astrocytes after Iba-1 and GFAP quantification, respectively [47].

Our study has some limitations. PD7 rat pups are considered to have an equivalent level of brain maturation to the 36-week gestation human neonate [48]. PD14 (as in this study) and PD49 animals exhibit a similar histopathological pattern [10,49], so PD14 can be used as a proper time-point to evaluate the neuroprotective efficacy of DMF. Although the neuroprotective effects have been partially addressed via histological techniques and imaging, further research is needed to unravel the molecular mechanisms by which DMF may induce its therapeutic response after neonatal HI. Further, if DMF has the ability to protect both differentiated neurons and neural stem/progenitor cells [50], it may also have the potential to treat asphyctic neonates through stimulation of endogenous neuroregeneration and plasticity.

## 5. Conclusions

Given that hypothermia offers limited protection in severe cases of newborn brain injury, we have used a severe model of neonatal hypoxia–ischemia to test the therapeutic effect of DMF. DMF induced a strong neuroprotective response following severe hypoxia–ischemia, evidenced by a reduction in brain infarct size and neuropathological score. Additionally, DMF decreased white matter brain injury by preserving myelin tracts and modulated the activation of microglia and astroglia.

## Data Availability

The data presented in this study are available on request from the corresponding author.
